# Enhancing Maize Yield and Soil Health through the Residual Impact of Nanomaterials in Contaminated Soils to Sustain Food

**DOI:** 10.3390/nano14040369

**Published:** 2024-02-16

**Authors:** Esawy Mahmoud, Asmaa El-shahawy, Mahmoud Ibrahim, Abd El-Halim A. Abd El-Halim, Atef Abo-Ogiala, Mohamed. S. Shokr, Elsayed Said Mohamed, Nazih Y. Rebouh, Sahar Mohamed Ismail

**Affiliations:** 1 Soil and Water Department, Faculty of Agriculture, Tanta University, Tanta 31111, Egypt; esawy.rezk@agr.tanta.edu.eg (E.M.); mahmoud.abouzaid@agr.tanta.edu.eg (M.I.);; 2 Water and Environment Research Institute, Sakha Agricultural Research Station, Kafr El-Sheikh P.O. Box 33717, Egypt; 3 Horticulture Department, Faculty of Agriculture, Tanta University, Tanta 31111, Egypt; atef.abo_ogiala@agr.tanta.edu.eg; 4 National Authority for Remote Sensing and Space Sciences, Cairo 1564, Egypt; 5 Department of Environmental Management, Institute of Environmental Engineering, RUDN University, 6 Miklukho-Maklaya, Moscow 117198, Russia; 6 Soil Physics and Chemistry Department, Desert Research Center, Cairo 4540031, Egypt

**Keywords:** nanomaterials, contaminated soil, residual effects, soil fertility, biological activity, maize

## Abstract

Studying the impact of residual soil nanomaterials is a promising challenge for sustainable agricultural development to improve soil health and crop productivity. The objective of this study is to assess the long-term impacts of 50, 100, and 250 mg kg^−1^ soil of nanobiochar (nB) and nano-water treatment residues (nWTR) on the fertility, biological activity, and yield of maize (*Zea mays* L.) growing in heavy metal-contaminated soils. The results showed that when nB and nWTR were added in larger quantities, the concentrations of lead (Pb), nickel (Ni), cadmium (Cd), and cobalt (Co) extracted with DTPA decreased. With the addition of nB or nWTR, it also showed a significant increase in exchangeable cations, cation exchange capacity (CEC), soil fertility, soil organic matter (OM), microbial biomass carbon (MBC), and a decrease in soil salinity and sodicity. Catalase and dehydrogenase activities rose as nB addition increased, while they decreased when nWTR addition increased. In comparison to the control, the addition of nB and nWTR greatly boosted maize yield by 54.5–61.4% and 61.9–71.4%, respectively. These findings suggest that the researched nanomaterials’ residual effect provides an eco-friendly farming method to enhance the qualities of damaged soils and boost maize production. Our research suggested that adding recycling waste in the form of nanoparticles could immobilize heavy metals, improve soil characteristics, and increase the soil’s capacity for productivity.

## 1. Introduction

Heavy metals and organic pollutants, which are defined by their toxicity, stability, non-degradability, and bioaccumulation, pollute the areas close to the factories. Since these places have an impact on output, soil quality, and public health, managing them may be one of the biggest problems [1]. The utilization of nanomaterials in agriculture is vital due to the recent increase in population and the loss of natural resources. Nanomaterials have a diameter of less than 100 nm and are characterized by a high surface area, high porosity, and active adsorption sites [2]. Nanomaterials have been applied to agriculture in recent years to boost crop yields and adsorb hazardous chemicals, including pesticides and heavy metals [3]. High surface area, high porosity, and more surface-active sites are characteristics of nanobiochar (nB) or nano-water treatment residue (nWTR), which promote improved nutrient retention and increased pollutant adsorption [1,4,5]. Enhancing the production of crops and effectively removing dangerous pollutants are two benefits of using nanobiochar. Additionally, it enhances the soil’s biological, chemical, and physical characteristics to make it more suitable for agricultural uses [6].

The effects of nB and nWTR addition on soil quality and canola yield in heavy clay soils were assessed by El-shahawy et al. [7]; the authors highlight the potential benefits of applying both nB and nWTR at 250 mg kg^−1^ soil to enhance soil fertility, aggregate stability, consistency, and canola yield. Zhou et al. [8] found that nB considerably changed the Loess Plateau’s soil moisture content and hypothesized that nB would lessen nitrogen loss due to erosion caused by rainfall. According to Wang et al. [9], nB fertilization lowered fertilizer use by 30% to 50% while increasing crop yields by 10% to 20%. The addition of nB and nWTR at varying rates in degraded soil resulted in a considerable increase in microbial biomass carbon (MBC), as well as the activities of dehydrogenase (DHA) and catalase (CLA) and the canola production in treated pots [7]. According to Mahdy et al. [10], stable As-nWTR surface complexes were formed by nWTRs, which effectively immobilized and adsorb arsenic (As) in agricultural soils modified with biosolids. According to earlier studies [1,11], nWTR can be used as a soil amendment to regulate P mobility and adsorption in salt-affected soils as well as in some heavy metals and dyes. The long-term benefits of organic materials on soil parameters can continue long after the treatment has ceased [12]. In the first year following application, Eghball and Power [13] discovered that 40% of the nitrogen from cattle dung and 20% from compost were available. This suggests that in the following few years, around 60% of the nitrogen from cattle manure and 80% from compost were available. According to Ramamurthy and Shivashankar [14], fertilizers, both organic and manufactured, applied to earlier crops have a major impact on the following crop and help maintain soil fertility. The residual effect of green manure may quadruple future grain production [15]. The production of the next wheat crop was greatly affected by the residual effect of farm manure [16]. Adding biochar to previous crops improved soil quality, yield metrics, and the productivity of the next crop of maize [17].

The adaptable crop maize (*Zea mays* L.) is utilized as animal feed and human nourishment and for a variety of industrial raw materials. After rice and wheat, it is regarded as Egypt’s third-most significant crop. Maize is grown on 1.03 million hectares in Egypt, representing about 25.2 percent of the total cultivated agricultural land, with an average productivity of 8.3 tons per hectare [18]. Increasing the soil’s sustainability and fertility is necessary to increase maize productivity. The novelty of this research is that it is a case study presenting the long-term effects of nB and nWTR applied at varying rates on the fertility, soil biology, and productivity of maize grown in soil contaminated with heavy metals. Therefore, the present study focused on the residual effect of nB and nWTR applied at varying rates on the maize yield and soil in soil contaminated with heavy metals.

## 2. Materials and Methods

### 2.1. Studied Area

**Figure 1:**
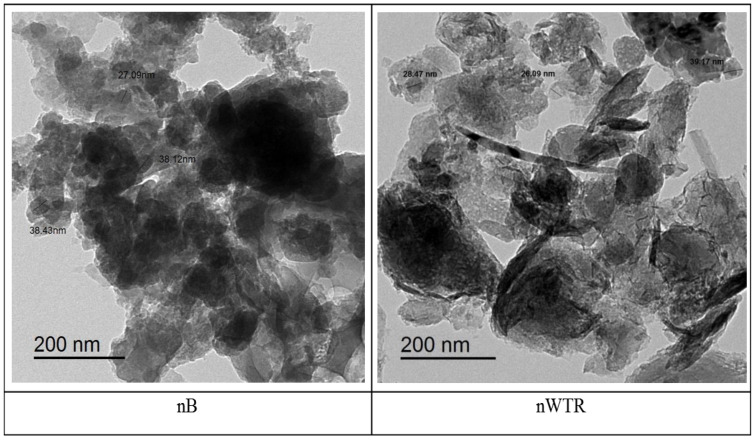
Transmission electron microscope (TEM) image for nanobiochar (nB) and nano-water treatment residuals (nWTR).

The Kafr El-Zayat area, at 30°49′31.68″ N latitude and 30°48′50.10″ E longitude, is contaminated with heavy metals. The average concentrations of Pb, Ni, Cd, Co, and Cr are 164.2, 1051.2, 26.6, 0.6, and 51.0 mg kg^−1^, respectively. These concentrations are higher than the allowable limits for agricultural soil established by US EPA [19]. The region is home to numerous sources of pollution, including the salt and soap industry, the superphosphate facility, and the pesticide factory. Vertic Torrifluvents (Entisols order) is the classification used for this location. Ten contaminated locations were chosen to have surface samples obtained at a depth of 0–20 cm. The manufacture and characterization of nanobiochar (nB) and nano-water treatment residues have been previously described by Elsawy et al. [5]. The analysis of the two investigated soils, nB and nWTR, used in the earlier pot experiment is displayed in Table 1. In Figure 1, the sizes of nB and nWTR are displayed.

### 2.2. Pot Experiment

At the Sakha Agricultural Research Station, Kafr El-Sheikh Governorate, Egypt, a pot experiment (diameter: 30 cm; height: 25 cm) that was previously used to test the direct contribution of additional nB and nWTR on polluted soil was employed for the study.

In this experiment, canola is a pre-maize crop, a winter crop that was planted on 5 October and harvested 22 weeks after planting. The prior study included nine treatments in a completely randomized experimental design, which were as follows: In control (C), soil without soil amendments; B, biochar at a rate of 4200 mg kg^−1^; nB_50_, nanobiochar at a rate of 50 mg kg^−1^; nB_100_, nanobiochar at a rate of 100 mg kg^−1^; nB_250_, nanobiochar at a rate of 250 mg kg^−1^; WTR, WTR at a rate of 4200 mg kg^−1^; nWTR_50_: NanoWTR at a rate of 50 mg kg^−1^; nWTR_100_: NanoWTR at a rate of 100 mg kg^−1^; and nWTR_250_, NanoWTR at a rate of 250 mg kg^−1^.

Maize (*Zea mays* L.), a test crop, was planted on 16 June 2021. The type of maize used was single hybrid 131. As in a prior study, the nB and nWTR were combined. After the seeds were deposited in each pot, 500 g of the same soil were collected, smoothed, well mixed, and passed through a sieve before being applied to the soil surface to guarantee a uniform distribution. Two hours after irrigation, it was added. To make sure the nB and nWTR were not washed away, water was then sprayed onto the soil until it reached 75% of the field capacity. Maize plants were thinned to two plants per pot two weeks after planting. Throughout the experiment, the temperature varied between 25 and 34 °C, and the relative humidity was between 45 and 60%. After being sown, the maize plants were harvested in September 2021, i.e., 102 days later. Plant materials were dried for 48 h at 75 °C in an oven to extract their dry matter. An electronic scale was used to measure the dry weight of the plant gravimetrically.

### 2.3. Analysis of Soil Samples and Nanobiochar

The Walkley–Black technique (1934) [20] was used for the OM test of soil, nWTR, and nB. The combustion method was utilized to estimate OM for B and nB [21]. While electrical conductivity (EC) was measured at a ratio of 1:10 (*w*/*v*) for all samples, pH was measured in soil (1:2.5), nB (1:10), and nWTR (1:2.5) using a pH meter and a conductivity meter, respectively. According to Olsen et al. (1954) [22], the available phosphorus (P) was extracted from the soil using a sodium bicarbonate solution (0.5 M, pH 8.5), ascertained by the ammonium molybdate method, and quantified using a spectrophotometer at 660 nm. The available potassium (K) was extracted using ammonium acetate and then measured using a flame photometer [23]. According to the Soil Survey Staff (2014) [24], the Kjeldahl method was utilized to measure the amount of available nitrogen (NH_4_^+^ and NO_3_^−^) that was removed from the soil by 2 M potassium chloride. According to [25], the bulk density of the undisturbed soil sample was determined using the core method. Ref. [26] states that ammonium acetate solution (1.0 mol L^−1^) with a pH of 7 was used to test the cation exchange capacity (CEC). The inductively coupled plasma optical emission spectrometry (ICP-OES) was used to measure the heavy metals removed using diethylenetriamine penta-acetic acid (DTPA) [27]. The measuring instrument is first calibrated with standard solutions, and the sample is analyzed. The ICP-OES characteristics were as follows: argon as the gas at 560 kPa, a power of 1.5 Kw, plasma at 9.99, accessory at 0.60, channel flow at 0.60, and a small purge flow. The rotating pump was adjusted to 45 rpm. and performed multiple times at a temperature of 34 °C, a CCD temperature of −10, and an impeller level of 1.5 Pa.

### 2.4. Catalase Activity

Catalase activity was measured by back-titrating leftover hydrogen peroxide (H_2_O_2_) with KMnO_4_ [28]. 2 g of soil samples was added to 40 mL of distilled water and 5 mL of a 0.3% H_2_O_2_ solution. The mixture was agitated for 20 min after the inclusion of 5 mL of 1.5 mol L^−1^ H_2_SO_4_. Following that, the solution was titrated with 0.02 mol L^−1^ KMnO_4_. The activity of catalase was estimated using the reacted quantity of 0.02 mol L^−1^ KmnO_4_ per g of dry soil [29].

### 2.5. Dehydrogenase Activity (DHA)

A 2 g quantity of air-dried soil and 2 mL of tetrazolium chloride were mixed, and the mixture was incubated for 24 h at 37 °C. This produced 2,3,5-triphenylformmazan (TPF) which was then extracted with 10 mL of acetone and measured at 485 nm to determine the DHA activity [30]. Using the conventional TPF curve, the following equation was utilized to calculate the activity of the enzymes:

Dehydrogenase activity µg TPF/g dry = (OD/K)/DW

where OD = optical density; DW = soil dry weight; and K = the factor obtained from the standard curve.

### 2.6. Microbial Biomass Carbon

Following a 24 h fumigation at 25 °C using ethanol-free chloroform, the MBC of 25 g of soil samples was evaluated [31]. Next, K_2_SO_4_ was used to extract the soil, and K_2_Cr_2_O_7_ and H_2_SO_4_ were used to measure the amount of extractable organic C after 30 min at 170 °C. Ferrous ammonium sulphate was finally used to titrate it, and ferroin was used as an indicator.

The following equation was used to calculate MBC:

(1) MBC = (EC fumigated soil − EC un − fumigated soil)/Kc

where EC = extractable carbon; and Kc = 0.379 (Kc is K_2_SO_4_ extraction efficiency), ref. [32].

### 2.7. Statistical Analysis

Using the SAS software (SAS 9.2), all acquired data were statistically examined. A statistical significance criterion of *p* < 0.05 was utilized to compare treatments using Duncan’s Multiple Domain Test (DMRT).

## 3. Results

### 3.1. Residual Effect of Nanomaterials on Remediation of Some Heavy Metals

**Figure 2:**
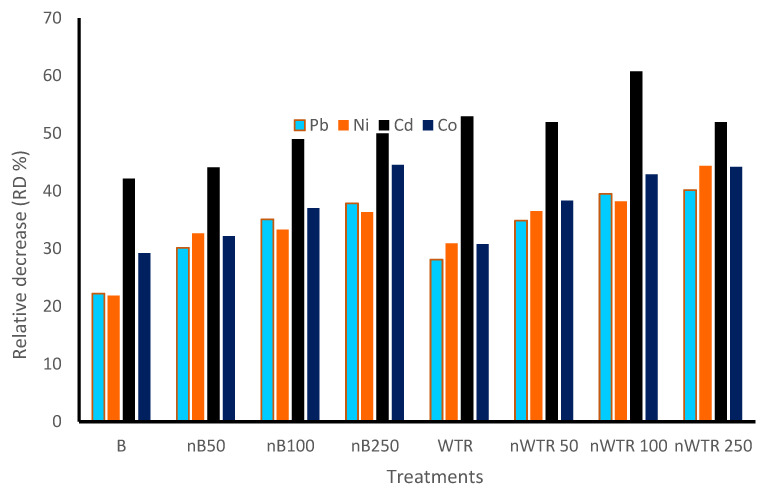
Residual effect of nanomaterials on remediation of some heavy metals.

The data presented in Table 2 demonstrated that all heavy metals under study (Pb, Ni, Co, and Cd) could be found in lower concentrations when using nanobiochar (nB) and nano-water treatment residuals (nWTR). However, the proportion of reduction differed among amendments. The relative decrease (RD%) in Pb, Ni, Co, and Cd contents was significantly impacted by the applications of the investigated soil amendment. The concentrations of Pb, Ni, Cd, and Co decreased by 22.21% and 28.13%, 21.87% and 30.92%, 42.15% and 52.49%, and 29.25% and 30.82%, respectively, upon the addition of B and WTR. The addition of 250 mg nWTR kg^−1^ to the dry soil produced the highest decrease in Pb, Ni, and Co extracted by DTPA. DTPA-extractable Pb, Ni, Cd, and Co concentrations decreased with the increasing addition of nB and nWTR. The relative decrease (%) in Figure 2 for Cd, was 44.11%, 49.01%, and 50.0% for nB_50_, nB_100_, and nB_250_, respectively, compared to the control treatment. The decrease in the concentrations of Pb, Ni, Cd, and Co extracted by DTPA in the nWTR-treated soils was higher than that in the nB-treated soils at the same rate.

### 3.2. Residual Effect of Nanomaterials on Some Soil Properties

The concentrations of cations and anions significantly decreased (*p* < 0.05) because of the residual effect of adding soil amendments at various levels (Table 3). The investigated soil’s EC ranged from 3.92 to 2.01 dSm^−1^; the soil treated with 250 mg WTR kg^−1^ had the lowest EC value, while the soil with the highest EC value was the one under study. In comparison to the control soil, the application of 50, 100, and 250 mg WTR kg^−1^ soil reduced the soil EC by 36.5, 44.4, and 48.7%, respectively.

Table 3 indicates that the pH values of the investigated soil amendments do not differ in a way that is statistically significant. The pH range of the soil was 7.65 to 7.89. The pH values of the soil were lower at 250 mg nB kg^−1^ soil than they were at 50 and 100 mg nB kg^−1^ soil. When nB_250_ was added, the pH dropped noticeably more than with the other treatments.

As shown in Table 3, the concentrations of cations and anions decreased significantly in pots treated with the residual effect of the addition of soil amendments at different levels as compared to the control. The application of the highest dose of nB (250 mg kg^−1^) decreased the Cl^−^, HCO_3_^−^, SO_4_^−−^ and Na^+^ concentrations by 47.1, 44.4, 45.6, and 47.2%, respectively, compared to the control. The addition of the nanomaterials to the soil resulted in an increase in the concentration of cations and anions relative to the control treatment. The cation and anion concentrations between nB and nWTR at various levels in this investigation were not statistically significant. Regarding SAR in soil, there was a noteworthy distinction between the treatments and the control; the range was 10.48 for the control and 6.86 for the WTR (Table 3). The SAR between most of the treatments in our analysis, except for treatment B, was not significant. With an increase in nWTR level, SAR readings dropped.

As presented in Table 4, the residual effect of adding soil nanomaterials on exchangeable Ca^++^, Mg^++^, and K^+^ was significantly increased, whereas Na^+^ decreased with their addition. Soil Ca^++^, Mg^++^, and K^+^ exchangeables increased with the increasing levels of nB and nWTR addition.

The difference in soil Na^+^ exchangeable between the levels of the nWTRs was not significant. The residual effect of the nWTR_250_ addition pot elevated the exchangeable Ca^++^ by 47.17% more than that of the control pot. Soil Ca^++^ exchangeable increased with the addition of nB_50_, nB_100_, and nB_250_ by 20.5%, 23.5%, and 47.7%, respectively, in comparison to the control treatment.

Soil CEC ranged from 43.09 cmol_c_ kg^−1^ in the control to 59.65 cmol_c_ kg^−1^ in nB_100_, with a significant difference seen between the various treatments (Table 4). CEC increased with the increasing rates of nWTR addition. The CEC increased with the application of nB_50_, nB_100_, and nB_250_ by 14.04, 38.43, and 23.48%, respectively, when compared to the control treatment. At the same rate, the CEC in the nB-amended pots was higher than that in the nWTR-amended pots. In this study, the CEC between B and nB, as well as nB_250_ and nWTR_250_, was not significant.

The residual effect of nanomaterials added to the soil at different rates showed a significant decrease in the ESP (Table 4). Soil ESP increased by 19.33%, 25.98%, and 23.85% for nB_50_, nB_100_, and nB_250_, respectively, in comparison to the control treatment. The addition of WTR gave the highest significant decrease in the ESP compared to other treatments.

Organic matter (OM) increased from 1.23% (C) to 1.56% for B and to 1.49% for WTR. A significant increase was seen when soil amendments were added at different levels (Table 5). The residual effect of the nWTR_250_ addition pot elevated the OM by 11.76% more than the control pot. In our study, OM was not significantly different between the various treatments. Nitrogen (N) and potassium (K) concentrations increased significantly in the pots altered with the application of the investigated nanomaterials. The concentration of N increased as the addition rate of nB increased, but there was no change in the concentration of P or K. The addition of WTR_100_ produced the highest and the most significant increase in the K concentration compared to other treatments.

### 3.3. Residual Effect of Nanomaterials on Soil Biological Activity

The residual effect of adding nanomaterials resulted in significant differences in the microbial biomass carbon (MBC) in soil, ranging from 165.7 mg kg^−1^ in the control to 295.1 mg kg^−1^ in the nWTR_50_ (Table 6). MBC increased by 1.31, 1.61, and 1.63 times with the addition of nB_50_, nB_100_, and nB_250_, respectively, in comparison to the control treatment. MBC increased with the increase in nB addition levels, whereas it decreased with the increase in nWTR.

As shown in Table 6, the activities of dehydrogenase (DHA) and catalase (CLA) increased significantly in the pots treated with the addition of the studied nanomaterials at different rates, except for B and nB_100_ for CLA. The activity of DHA increased with the increase in nB addition levels, whereas it decreased with the increase in nWTR. The highest DHA and CLA activities were observed in nWTR-treated soils at 50 mg kg^−1^, with increases of 39.3% and 350% relative to the control, respectively. Catalase activity rose by 250% in the soil treated with nB_50_, rising from 0.04 mL KMnO_4_ per g of dry soil for the control to 0.14 mL KMnO_4_ per g of dry soil. It increased by 225% over the control treatment in soil treated with WTR_250_. No significant differences in DHA and CLA activities were observed among all treatments, except for nWTR50.

### 3.4. Residual Effect of nB and nWTR on Maize Growth

**Figure 3:**
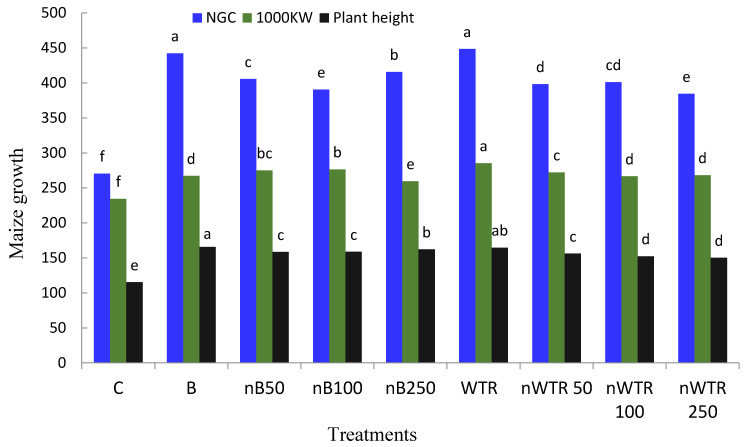
Residual effect of soil amendments on plant height (cm), number of grains per cob (NGC), and 1000 kernels weight (g) (100 KW) of maize crop. Control (C): soil without soil amendments; B: biochar at a rate of 4200 mg kg^−1^; nB50: nanobiochar at a rate of 50 mg kg^−1^; nB100: nanobiochar at a rate of 100 mg kg^−1^; nB250: nanobiochar at a rate of 250 mg kg^−1^; WTR: WTR at a rate of 4200 mg kg^−1^; nWTR 50: NanoWTR at a rate of 50 mg kg^−1^; nWTR100: NanoWTR at a rate of 100 mg kg^−1^, and nWTR250: NanoWTR at a rate of 250 mg kg^−1^. The same letter indicate no significant differences (*p* ≤ 0.05).

The addition of soil amendments at varying rates resulted in a considerable increase in the number of rows per cob and plant height of maize (Figure 3). In the WTR treatment, maize plants’ plant height and number of rows per cob increased from 11.1 and 115.4 cm in the control to 16.9 and 164.7 cm, respectively. When nB increased, the number of rows per cob and plant height of maize plants increased, but when nWTR increased, they dropped.

There was a noticeable difference in the treatments, as evidenced by the grains per cob and 1000 kernel weights of the maize plants, which varied from 270.5 and 234.5 g in the control to 448.5 and 285.4 g in the WTR (Figure 3). In comparison to the control treatment, the number of grains per cob of maize plants increased by 49.94%, 44.39%, and 53.72% for nB50, nB100, and nB250, respectively.

The addition of nB and nWTR had a significant effect on the grain yield of maize crops grown on degraded soil, as shown in Table 6. Maize yield increased by 54.5–61.4% and 61.91% for the nB and nWTR-amended soils, respectively. A significantly higher grain yield of maize was obtained by the WTR treatment as compared to that with the control treatment. No significant difference in the grain yield of the maize crop was observed among all treatments, except for WTR.

## 4. Discussion

### 4.1. Residual Effect of Nanomaterials on Immobilization of Some Heavy Metals

The decrease in extractable Pb, Ni, Cd, and Co concentrations in WTR-amended soils may be related to the higher clay content, CEC, and OM of WTR (Table 1). Additionally, the nWTR used in the study contained 2500, 111.2, and 66.0 mg kg^−1^ of aluminum (Al), Ca, and Mg, respectively. This indicates the presence of the Al, Ca, and Mg components, which may be involved in the adsorption of the studied heavy metals. The increase in the immobilization of heavy metals in the soil treated with nWTR, Pb, Ni, Cd, and Co concentrations could be attributed to the formation of surface mineral complexes. These complexes may be formed due to the interaction of the metal with the surface sites of oxides such as Al-OH, Si-OH, and Ca-OH found in the nWTR. In a previous study by El-Sawy et al. [5], demonstrated that the high surface areas of nWTR (114.33 m^2^ g^−1^) and nB (289.57 m^2^ g^−1^) were important for the adsorption of large amounts of organic and inorganic pollutants [33,34]. The zeta potentials were −31.08 mV and −65.25 mV for nB and nWTR, respectively, which means that there are negative charges on their surfaces, which serve to adsorb heavy metals [35]. The functional groups on nB and nWTR can act as adsorption sites for heavy metals through electrostatic interaction, ion exchange [5,36,37], and surface complexity [38,39]. Metals such as Ni, Cd, Co, and Pb can serve as a bridge between nB or nWTR and clay minerals [40].

### 4.2. Residual Effect of nB and nWTR on Soil Properties

The decrease in ESP and EC in soil treated with residual nB or nWTR corresponded with improved soil physical properties [7]. Ca^++^, K^+^, and Mg^++^ released from biochar displaced Na^+^ from exchangeable sites in saline soil, enhancing Na^+^ leaching out of the soil profile [41]. ESP was reduced in saline soils as a result of biochar addition [42]. Huang and Gu [43] found a significant decrease in the EC of soil from 13.8% to 36.5% with the addition of woodchip biochar compared to untreated soil. Soil amended with biochar improves porosity and hydraulic conductivity, resulting in faster salt removal and reduced soil salinity (EC) [44]. Biochar amendment improves soil structure and porosity, which enhances Na leaching and leads to lower ESP for saline-sodic soils [45]. The increase in CEC in the residual nB is due to some residual biochar particles that have negative charges, which increase its absorption capacity and basic saturation in the soil environment [46]. The resistance of the remaining nB to the organisms leads to carbon availability in the soil environment and thus works to increase the cation exchange sites [46,47]. In this study, we proposed two mechanisms that demonstrate an increase in CEC with the addition of nB or nWTR: (1) the higher surface area of nB or nWTR increases the adsorption of cations and (2) nB or nWTR has a high zeta potential that stabilizes cations on its surface.

The sluggish release of N into the soil N pool is probably caused by the residual NH4+ adsorbed on the nB and nWTR surfaces from the previously supplied NPK, which accounts for the increased N content [48,49,50]. nB has been shown to interact with microbial communities in the rhizosphere, enhancing soil nutrient availability through microbial activity and mineralization, due to its high surface area as well as its ability to exchange cations [51]. The slow mineralization of the residual nB led to the release of non-volatile nutrient cations, which caused an increase in the contents of Mg^++^ and Ca^++^ [52].

### 4.3. Residual Effect of nB and nWTR on Enzymatic Activity

The study found that adding nB improved the chemical and physical characteristics of the residual nB-amended soil, and its higher contents of organic matter, nutrients, and CEC contributed to an increase in DHA, CLA, and MBC (Table 1). These results are in line with those of [53], who found a relationship between soil fertility, organic matter, and long-term productivity and enzyme activity. According to [54], the addition of organic materials boosted the microbial biomass and enzymatic activity of the soil by increasing the number of microorganisms in the soil. Microbial biomass (C, DHA, and CLA) increased with the increase in the addition rates of residual nB, whereas it decreased with the increase in nWTR. Microbial biomass carbon (MBC), DHA, CLA, and maize production significantly increased in pots treated with different amounts of nB and nWTR in degraded soil. Because nWTR improves the biochemical and physical qualities of the soil and increases its amounts of nutrients, clay, and organic matter, it increases enzymatic activity and MBC in soils treated with it (Table 1). Because aluminum is toxic in high concentrations, the addition of leftover nWTR in soil at a rate greater than 50 mg kg−1 resulted in a drop in MBC and enzymatic activity.

### 4.4. Residual Effect of nB and nWTR on Maize Growth

Increased grain yield in pots containing residual nanomaterials corresponded to soil fertility, MBC, CEC, and organic matter [4,5].When compared to the control treatment, the preceding crops that had nanomaterials added to them produced the highest grain yield (3199 kg ha^−1^). Previous research [42,55] has examined the impacts of residual biochar on crops, such as wheat and rice, and how they boost the yield of crops. The availability of nutrients, some root remnants from the previous crop, and a growth-friendly environment are all responsible for the increase in maize production observed in our study across all treatments [47]. This rise is consistent with the soil’s chemical composition and increased enzymatic activity. According to [56], the presence of nanomaterials may have an impact on plant performance by enabling plant microbial assembly, inhibiting pathogens, enhancing soil nutrient availability, and mineralizing leftover organic matter. According to [5,57], they found a strong positive correlation between the dry weights of canola plants and MBC (R^2^ = 0.80), CEC (R^2^ = 0.72), and OM (R^2^ = 0.83). The dry weight of the maize plant decreased as WTR grew because Al-PO4 and Ca-PO4 production lowered the amount of available P. It is common knowledge that P affects plant development. According to the findings, the high concentrations of nWTR reduce soil enzyme activity and canola plant productivity in the soils under investigation. These findings could be a crucial sign for limiting the application of nanomaterials in agriculture.

## 5. Conclusions

In this study, we showed that the concentrations of Pb, Ni, Cd, and Co extracted with DTPA decreased with the increasing addition of nB and nWTR. The relative decrease (%) in Cd was 44.11%, 49.01%, and 50.0% for nB_50_, nB_100_, and nB_250_, respectively, compared to the control treatment. The results showed that the residual effects of nB and nWTR reduced soil salinity and sodicity and improved soil fertility. The activities of dehydrogenase and catalase, OM, CEC, and MBC increased with the increase in the addition levels of nB. The addition of residual nWTR at a rate greater than 50 mg kg^−1^ soil led to a decrease in enzymatic activity, MBC, and maize yield due to the toxicity of aluminum at high levels. The study’s conclusions suggest that the residual effects of nWTR and nB should be taken into account when adding organic and inorganic fertilizers, maintaining sustainable development, and restoring degraded soils. 

## Figures and Tables

**Table 1:** Properties of soil, nB, and nWTR used in the previous experiment.

Characteristics	Units	Soil	nB	nWTR
Sand	%	12.56	-	-
Silt		28.26	-	-
Clay		59.18	-	68.6
Soil texture		clay	-	-
pH		7.84	8.24	7.49
EC	dSm^−1^	3.78	2.45	1.12
Ca^++^	meqL^−1^	7.9	55.11	5.56
Mg^++^		4.5	24.30	5.50
K^+^		0.57	-	-
Na^+^		25.7	-	-
Cl^−^		18	-	-
HCO_3_^−^		2.5	-	-
SO_4_^−−^		18.3	-	-
SAR		10.29	-	-
OM	%	1.51	49.8	4.82
CEC	cmol(+) kg^−1^		31.3	38.85
Total N	%	-	1.42	0.98
Total P	%	-	0.96	0.06
Total K	%	-	1.42	0.65
Total Al	%	-	-	0.25
Available P	mg kg^−1^	45.6	856.2	14.46

**Table 2:** Effect of soil amendments on immobilization of bioavailable heavy metals.

Treatment	Pb	Relative Decrease (RD %) of Pb	Ni	Relative Decrease (RD %) of Ni	Cd	Relative Decrease (RD %) of Cd	Co	Relative Decrease (RD %) of Co
C	62.12 ^a^		12.48 ^a^		0.204 ^a^		14.63 ^a^	
B	48.32 ^b^	22.21	9.75 ^b^	21.87	0.118 ^b^	42.15	10.35 ^b^	29.25
nB_50_	43.38 ^cd^	30.16	8.4 ^bc^	32.69	0.114 ^b^	44.11	9.92 ^bc^	32.19
nB_100_	40.32 ^def^	35.09	8.32 ^bc^	33.33	0.104 ^b^	49.01	9.21 ^bcd^	37.04
nB_250_	38.59 ^ef^	37.87	7.94 ^bc^	36.37	0.102 ^b^	50	8.11 ^d^	44.56
WTR	44.64 ^c^	28.13	8.62 ^bc^	30.92	0.096 ^b^	52.94	10.12 ^b^	30.82
nWTR_50_	40.46 ^de^	34.86	7.92 ^c^	36.53	0.098 ^b^	51.96	9.02 ^bcd^	38.34
nWTR_100_	37.57 ^ef^	39.52	7.71 ^c^	38.22	0.08 ^b^	60.78	8.35 ^cd^	42.92
nWTR_250_	37.17 ^f^	40.16	6.94 ^c^	44.39	0.098 ^b^	51.96	8.16 ^d^	44.22
F-test	**		**		**		**	
LSD_(0.05)_	2.387		1.35		0.032		1.182	
LSD_(0.01)_	3.271		1.815		0.044		1.621	

Control (C): soil without soil amendments; B: biochar at a rate of 4200 mg kg^−1^; nB_50_: nanobiochar at a rate of 50 mg kg^−1^; nB_100_: nanobiochar at a rate of 100 mg kg^−1^; nB_250_: nanobiochar at a rate of 250 mg kg^−1^; WTR: WTR at a rate of 4200 mg kg^−1^; nWTR _50_: Nano-WTR at a rate of 50 mg kg^−1^; nWTR_100_: Nano-WTR at a rate of 100 mg kg^−1^, and nWTR_250_: Nano-WTR at a rate of 250 mg kg^−1^. Note: values of each now followed by the same letter indicate no significant differences (*p* ≤ 0.05) according to Duncan test and NS: Not significant, **: High significant.

**Table 3:** Residual effect of nanomaterials on some soil chemical properties.

Treatment	EC	pH	SAR	Anions			Cations			
				Cl^−^	HCO_3_^−^ meq L^−1^	SO_4_^−−^	Ca^++^	Mg^++^	Na^+^ meq L^−1^	K^+^
C	3.92 ^a^	7.89 ^a^	10.48 ^a^	18.70 ^a^	4.50 ^ab^	16.90 ^a^	8.20 ^a^	4.70 ^a^	26.70 ^a^	0.53 ^b^
B	3.08 ^ab^	7.68 ^a^	9.51 ^ab^	14.70 ^b^	2.50 ^c^	14.06 ^b^	6.50 ^b^	3.70 ^ab^	20.90 ^b^	0.82 ^a^
nB_50_	2.17 ^bc^	7.74 ^a^	7.80 ^bc^	10.30 ^c^	2.50 ^c^	9.60 ^c^	4.60 ^c^	2.60 ^bc^	14.80 ^c^	0.53 ^b^
nB_100_	2.03 ^c^	7.79 ^a^	7.43 ^c^	9.40 ^c^	2.50 ^c^	8.70 ^cd^	4.10 ^c^	2.40 ^c^	13.40 ^c^	0.66 ^ab^
nB_250_	2.18 ^bc^	7.65 ^a^	7.63 ^c^	9.90 ^c^	2.50 ^c^	9.20 ^c^	4.40 ^c^	2.50 ^c^	14.10 ^c^	0.62 ^ab^
WTR	2.65 ^bc^	7.67 ^a^	6.86 ^c^	9.40 ^c^	3.00 ^bc^	8.50 ^cd^	4.10 ^c^	2.40 ^c^	13.40 ^c^	0.49 ^b^
nWTR _50_	2.49 ^bc^	7.71 ^a^	8.60 ^bc^	10.60 ^c^	3.00 ^bc^	9.40 ^c^	4.30 ^c^	2.10 ^c^	15.40 ^c^	0.55 ^b^
nWTR _100_	2.18 ^bc^	7.68 ^a^	7.82 ^bc^	10.40 ^c^	5.00 ^a^	7.20 ^d^	4.60 ^c^	2.60 ^bc^	14.80 ^c^	0.59 ^b^
nWTR _250_	2.01 ^c^	7.69 ^a^	6.94 c	8.90^c^	2.50 ^c^	7.10 ^d^	3.60 ^c^	2.10 ^c^	11.70 ^c^	0.63 ^ab^
F-test	**	NS	**	**	**	**	**	**	**	*
LSD_(0.05)_	0.678	1.821	1.541	3.039	1.357	1.715	1.443	0.958	3.470	0.156
LSD_(0.01)_	0.93	2.49	2.11	4.16	1.86	2.35	1.97	1.31	4.75	0.21

Note: values of each now followed by the same letter indicate no significant differences (*p* ≤ 0.05) according to Duncan test and NS: Not significant, *: Significant; **: High significant.

**Table 4:** Residual effect of nanomaterials on exchangeable cations.

Treatments	Exchangeable Cations (cmol kg^−1^)				CEC cmol kg^−1^	ESP
	Ca^++^	Mg^++^	Na^+^	K^+^		
C	20.88 ^d^	10.91 ^de^	9.52 ^a^	1.14 ^cd^	43.09 ^f^	22.09 ^a^
B	26.52 ^bc^	11.37 ^de^	9.05 ^b^	1.08 ^d^	48.92 ^cde^	18.49 ^b^
nB_50_	25.16 ^c^	11.71 ^bcd^	8.76 ^c^	1.43 ^ab^	49.14 ^cde^	17.82 ^bc^
nB_100_	25.78 ^c^	13.33 ^a^	8.12 ^d^	1.35 ^ab^	59.65 ^a^	16.35 ^c^
nB_250_	30.84 ^a^	11.28 ^de^	8.95 ^bc^	1.46 ^a^	53.21 ^b^	16.82 ^bc^
WTR	28.61 ^ab^	10.63 ^e^	6.14 ^f^	1.34 ^ab^	47.03 ^e^	13.05 ^d^
nWTR_50_	27.13 ^bc^	12.46 ^b^	6.83 ^e^	1.26 ^bc^	48.62 ^de^	14.04 ^d^
nWTR_100_	29.42 ^a^	11.62 ^cd^	7.06 ^e^	1.37 ^ab^	49.83 ^cd^	14.16 ^d^
nWTR_250_	30.73 ^a^	12.28 ^bc^	6.87 ^e^	1.41 ^ab^	51.46 ^bc^	13.35 ^d^
F-test	**	**	**	**	**	**
LSD_(0.05)_	1.632	0.816	0.284	0.171	2.018	1.295
LSD_(0.01)_	2.236	1.118	0.390	0.235	2.765	1.774

Note: values of each now followed by the same letter indicate no significant differences (*p* ≤ 0.05) according to Duncan test and NS: Not significant, **: High significant.

**Table 5:** Residual effect of nanomaterials on soil organic matter (OM) and available nutrients.

Treatment	O.M %	Available Nutrients		
		N	P mg kg^−1^	K
C	1.23 ^c^	47.57 ^d^	41.7 ^a^	273 ^e^
B	1.56 ^a^	59.5 ^a^	45.6 ^a^	358.8 ^abcd^
nB_50_	1.49 ^ab^	52.5 ^bcd^	44.4 ^a^	304.2 ^de^
nB_100_	1.47 ^ab^	57.75 ^ab^	43.3 ^a^	421.2 ^ab^
nB_250_	1.31 ^bc^	56 ^abc^	43.8 ^a^	390 ^abc^
WTR	1.49 ^ab^	54.25 ^abc^	41.6 ^a^	343.2 ^bcde^
nWTR_50_	1.42 ^abc^	52.5 ^bcd^	45.6 ^a^	327.6 ^cde^
nWTR_100_	1.46 ^abc^	54.25 ^abc^	43.4 ^a^	436.8 ^a^
nWTR_250_	1.32 ^bc^	50.75 ^cd^	41.8 ^a^	374.4 ^abcd^
F-test	*	**	NS	**
LSD_(0.05)_	0.242	4.502	4.401	58.267
LSD_(0.01)_	0.332	6.168	6.03	79.831

Note: values of each now followed by the same letter indicate no significant differences (*p* ≤ 0.05) according to Duncan test and NS: Not significant, *: Significant; **: High significant.

**Table 6:** Residual effect of nanomaterials on soil microbial biomass carbon (MBC), dehydrogenase activity (DHA), catalase activity (CLA), and grain yield of maize crop.

Treatments	Grain Yield t ha^−1^	MBC mg kg^−1^	DHA mg TPF/g Dry Soil	CLA (mL of 0.02 mol/L KMnO_4_ g^−1^)
C	1.89 ^c^	165.7 ^f^	0.56 ^d^	0.04 ^g^
B	3.54 ^ab^	265.8 ^b^	0.66 ^c^	0.06 ^fg^
nB_50_	3.05 ^b^	218.3 ^cd^	0.71 ^bc^	0.14 ^bc^
nB_100_	2.92 ^b^	267.4 ^b^	0.69 ^bc^	0.08 ^efg^
nB_250_	3.01 ^b^	271.6 ^b^	0.74 ^b^	0.13 ^bcd^
WTR	3.81 ^a^	224.9 ^c^	0.68 ^c^	0.11 ^cde^
nWTR_50_	3.24 ^ab^	295.1 ^a^	0.78 ^a^	0.18 ^a^
nWTR_100_	3.18 ^ab^	203.5 ^e^	0.69 ^bc^	0.16 ^b^
nWTR_250_	3.06 ^b^	204.9 ^de^	0.67 ^c^	0.09 ^def^
F-test	**	**	**	**
LSD_(0.05)_	0.520	10.758	0.042	0.031
LSD_(0.01)_	0.712	14.739	0.057	0.043

Note: values of each now followed by the same letter indicate no significant differences (*p* ≤ 0.05) according to Duncan test and NS: Not significant, **: High significant

## Data Availability

All data are included in the manuscript.
